# Mosquito age and avian malaria infection

**DOI:** 10.1186/s12936-015-0912-z

**Published:** 2015-09-30

**Authors:** Romain Pigeault, Antoine Nicot, Sylvain Gandon, Ana Rivero

**Affiliations:** MIVEGEC, UMR CNRS, 5290 Montpellier, France; CEFE, UMR CNRS, 5175 Montpellier, France

**Keywords:** Immune senescence, Ecological immunity, *Culex*, Avian malaria, Haemocyte

## Abstract

**Background:**

The immune system of many insects wanes dramatically with age, leading to the general prediction that older insects should be more susceptible to infection than their younger counterparts. This prediction is however challenged by numerous studies showing that older insects are more resistant to a range of pathogens. The effect of age on susceptibility to infections is particularly relevant for mosquitoes given their role as vectors of malaria and other diseases. Despite this, the effect of mosquito age on *Plasmodium* susceptibility has been rarely explored, either experimentally or theoretically.

**Methods:**

Experiments were carried out using the avian malaria parasite *Plasmodium relictum* and its natural vector in the field, the mosquito *Culex pipiens.* Both innate immune responses (number and type of circulating haemocytes) and *Plasmodium* susceptibility (prevalence and burden) were quantified in seven- and 17-day old females. Whether immunity or *Plasmodium* susceptibility are modulated by the previous blood feeding history of the mosquito was also investigated. To ensure repeatability, two different experimental blocks were carried out several weeks apart.

**Results:**

Haemocyte numbers decrease drastically as the mosquitoes age. Despite this, older mosquitoes are significantly more resistant to a *Plasmodium* infection than their younger counterparts. Crucially, however, the age effect is entirely reversed when old mosquitoes have taken one previous non-infected blood meal.

**Conclusions:**

The results agree with previous studies showing that older insects are often more resistant to infections than younger ones. These results suggest that structural and functional alterations in mosquito physiology with age may be more important than immunity in determining the probability of a *Plasmodium* infection in old mosquitoes. Possible explanations for why the effect is reversed in blood-fed mosquitoes are discussed. The reversal of the age effect in blood fed mosquitoes implies that age is unlikely to have a significant impact on mosquito susceptibility in the field.

**Electronic supplementary material:**

The online version of this article (doi:10.1186/s12936-015-0912-z) contains supplementary material, which is available to authorized users.

## Background

Ever since Manson [1] and Ross [2] demonstrated over a century ago that some of the key human diseases are not transmitted through contaminated air or water, but through the bite of infected mosquitoes, a great deal of effort has been invested in identifying the genetic and environmental determinants of mosquito competence for these parasites. Mosquito immunity has taken centre stage in these efforts. Laboratory gene silencing studies have provided key insights into immune pathways that limit the prevalence and intensity of parasitic infections in mosquitoes [3–5]. More recently, the bourgeoning field of ecological immunology has shifted the attention to the causes and consequences of variation in immune function in insects and, in particular, to the role played by non-genetic factors such as temperature [6], nutrition [7], reproductive status [8, 9], and previous infectious history [10, 11].

One of the best documented sources of heterogeneity in the immune system of multicellular organisms is their age. In virtually all species, immune function decreases drastically with age, a process that has been termed immune senescence and for which both mechanistic and evolutionary explanations have been proposed [12, 13]. Insects are no exception to this rule. Significant decreases in the antibacterial activity, melanization potential and number of haemocytes with age have been reported in a range of widely different insect taxa, including bees [14–16], butterflies [17, 18], scorpionflies [19], crickets [20], damselflies [21], flies [22, 23], and also mosquitoes [22–27].

The ubiquity of immune senescence leads to the general prediction that older insects should be more susceptible to infection than their younger counterparts, i.e., that individuals exposed to parasites later in life should have a higher probability and/or intensity of infection. This prediction has been confirmed in some cases, but not in others. Hillyer et al. [25] and Roberts and Hughes [28] both found that, as expected, a decrease in immunity with age is associated with an increase in parasite susceptibility. Surprisingly, however, in many insect species individuals seem to become more resistant to parasites as they age (see Additional file 1). Unfortunately, however, few of these studies have concomitantly measured immune function and parasite susceptibility, so the potential role played by the immune system cannot be established.

Understanding the effects of mosquito age on parasite transmission is particularly relevant for malaria. Early on, MacDonald [29] used mathematical epidemiology to show that vector lifespan is one of the most important determinants of malaria transmission. Indeed, the time lag between adult emergence and the activation of the host-seeking behaviour [30] combined with the long extrinsic incubation period of *Plasmodium* (approximately 14 days), entail that infectious mosquitoes are, necessarily, old. Consequently, old mosquitoes are often considered to be epidemiologically more important vectors of malaria than their younger counterparts. The age structure of the mosquito population also has important applied implications because it has been suggested that effective malaria control, with only weak selection for insecticide resistance, could be achieved by using insecticides that target only old mosquitoes [31, 32]. Yet, aside from a few studies that have shown that age-dependent mortality could affect malaria epidemiology [33–35], most epidemiological models of malaria do not take age structure explicitly into account [36]. Specifically, the potential for age-dependent susceptibility to *Plasmodium* in mosquitoes has been largely overlooked. Yet, early studies showed that *Aedes aegypti* mosquitoes become less susceptible to a *Plasmodium gallinaceum* infection as they age [37]. This effect challenges the claim that old females are more important vectors of *Plasmodium* and could have far reaching consequences for malaria transmission. Further experimental studies monitoring the effects of mosquito age on immunity and susceptibility to *Plasmodium* parasites are therefore required to evaluate the importance of mosquito age-structure on malaria epidemiology.

Here, the effect of age on both mosquito immunity and susceptibility to a *Plasmodium* infection are quantified using an experimental system consisting on the avian malaria parasite *Plasmodium relictum* and its natural vector in the field, the mosquito *Culex pipiens.* This parasite is ubiquitous in natural populations of birds [38] and is known to incur an important fecundity cost on their vectors [39]. The aim of this study is three-fold. First to establish the existence of immune senescence in *Cx pipiens* by quantifying the number of circulating haemocytes in the haemolymph of three, seven and 17-day old mosquitoes. Haemocytes are a crucial component of the mosquito immune system that kill pathogens via phagocytic, lytic and melanization pathways [40] and play a key role in the defence against *Plasmodium* [41], although the precise relationship between haemocyte density and *Plasmodium* protection has not been established, and may not necessarily be linear. Second, to establish whether changes in haemocyte numbers with age are associated with changes in the susceptibility to a *Plasmodium* infection: are old mosquitoes more or less susceptible to a *Plasmodium* infection? And third, to establish whether the effect of age on either the immune function or parasite susceptibility is modulated by the previous blood feeding history of the mosquitoes. Indeed, in practice, old mosquitoes are likely to have taken at least one previous uninfected blood meal. Previous blood meals drastically alter the physiology of the mosquitoes [42] and could potentially reset the effect of age on susceptibility to malaria [37, 43].

## Methods

### *Plasmodium* strain and bird infections

*Plasmodium relictum* (lineage SGS1) is the aetiological agent of the most prevalent form of avian malaria in Europe [44]. The biology of avian malaria is similar to that of human malaria, both in the vertebrate host and in the mosquito, which is why avian malaria has historically played a key role in human malaria research [45]. The lineage was isolated from infected sparrows in 2009 [45] and passaged to naïve canaries (*Serinus canaria)*. Since then it has been maintained by carrying out regular passages between the stock canaries through intraperitoneal injections. Experimental canaries (n = 5) were infected by injecting them with ca. 80 µL of blood from the infected canary stock. Mosquito blood feeding took place 12 days after the injection, to coincide with the acute phase of the *Plasmodium* infection [46].

### Oocyst prevalence and burden

**Fig. 1:**
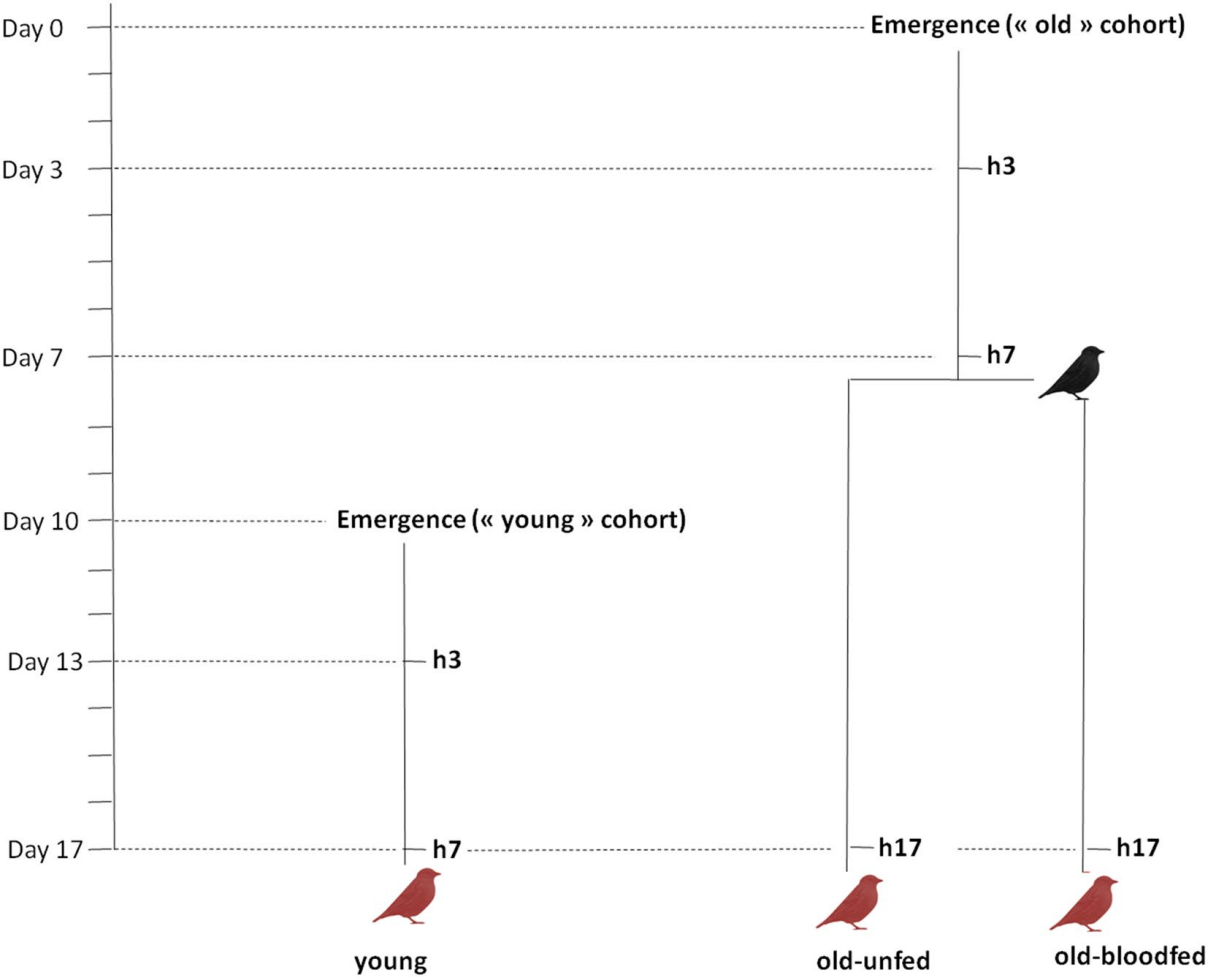
Schematic representation of the experimental design. *Black bird* uninfected blood meal; *red birds* *Plasmodium*-infected blood meal; h3, h7, h17: haemolymph sampling from 3, 7 and 17-day old mosquitoes, respectively. Haemolymph was sampled immediately before the blood meal on a separate sample of mosquitoes (haemocyte extraction is destructive). Young, old-unfed and old-blood fed mosquitoes are *colour* marked and fed from the same bird (n = 5 different birds)

Experiments were carried out using a laboratory line of *Cx pipiens* (SLAB, [46]). The experimental design is shown in Fig. 1. To test the effect of mosquito age on the probability and intensity of a *Plasmodium* infection, two mosquito cohorts were generated: ‘old’ (17-day old) mosquitoes (which was later split between ‘blood fed’ and ‘unfed’, see below) and ‘young’ (7-day old) mosquitoes. The larvae for both of these cohorts were raised in an identical way following previously published protocols [46]. Larval trays (n = 6 for the young and n = 10 for the old cohorts) were placed individually inside an ‘emergence cage’ (40 × 28 × 31 cm) and emerged adults were allowed to feed ad libitum on a 10 % glucose water solution.

The experiment proceeded as follows. For the old cohort, on the day of emergence (Day 0, Fig. 1) mosquitoes were haphazardly sampled from the emergence cages (*c.a.* 20 mosquitoes from each emergence cage) and placed in six different experimental cages. Seven days later (Day 7) the cages were randomly allocated to either the ‘old-unfed’ treatment or the ‘old-blood fed’ treatment, and the latter were provided with an uninfected canary to blood feed (a different canary per cage) following previously published protocols [46]. Blood feeding success was confirmed through the visual inspection of the mosquito abdomen. Unfed mosquitoes (<7 %) were discarded. The young cohort was launched 10 days after the old cohort (Day 10, Fig. 1) in an identical way. All mosquitoes were given a *Plasmodium*-infected blood meal on Day 17. For this purpose, 50 mosquitoes from each of the three experimental treatments (young, old-unfed, old-blood fed) were placed together inside a cage (n = 5 different cages) and allowed to blood feed from a *Plasmodium*-infected bird for 12 h. To distinguish between the three treatments, mosquitoes were previously marked using three different fluorescent colour powders and the colour allocated to each treatment changed in each of the cages (for details, see [47]).

To obtain an estimate of blood meal size, 1 day after the infected blood meal, all blood-engorged females were placed individually in numbered plastic tubes (30 ml) covered with a mesh. Food was provided in the form of a cotton pad soaked in a 10 % glucose solution. Seven days later (Day 7 post-blood meal) the females were taken out of the tubes and amount of haematin excreted at the bottom of each tube was quantified as an estimate of the blood meal size [46]. Females were then dissected and the number of *Plasmodium* oocysts in their midguts counted with the aid of a binocular microscope [46]. The wings of ten haphazardly chosen females from each size and treatment combination were removed prior to the midgut dissection and measured along its longest axis as an index of body size.

As the young and old cohorts are not launched simultaneously, there is a risk is that small differences in the larval rearing conditions may result in adults that varied in their body condition independently of their age, thereby potentially confounding the results. To account for this, the entire experiment was repeated 4 months later (the experiments are hereafter called Blocks 1 and 2).

### Haemolymph collection and haemocyte quantification

To establish whether the patterns of oocyst prevalence and burden in old and young mosquitoes are correlated with their immune activity, a sub-sample of ten to 15 mosquitoes from each cage and treatment combination was taken at regular intervals throughout the experiment and their haemolymph extracted to count the number of circulating haemocytes. The soft exoskeleton of newly emerged mosquitoes made the task of sampling the haemolymph on the day of emergence impossible. Old and young mosquitoes were therefore sampled 3 days after emergence (once their exoskeleton had hardened) and immediately before each of the blood meals: on Days 7 (old mosquitoes only) and 17 (old and young mosquitoes) of the experiment (see Fig. 1). The wing of haphazardly sampled females from each cages were measured as above to control for body size, and haemolymph were extracted individually following the protocols described below.

The haemolymph collection protocol was adapted from one described by Qayum and Telang [48]. Mosquitoes were injected between the seventh and eighth abdominal segments with an anticoagulant solution (70 % Schneider’s Insect medium and 30 % citrate buffer) and placed on ice for 5 min to allow the anticoagulant solution to dislodge haemocytes adhering to the internal tissues. The tip of the abdomen was then removed and, using a Hamilton™ syringe, 10 µL of anticoagulant buffer was injected in the lateral side of mosquito mesothorax. This caused the diluted haemolymph to flow out of the abdomen. Eight microlitre of diluted haemolymph were collected with a pipette equipped with a sterile tip and placed on a glass slide. Slides were placed in the dark for 20 min to allow the haemocytes to adhere to the slide’s surface. The haemocytes were then fixed and stained using the Microscopy Hemacolor^®^ staining kit as follows. Each glass slide was dipped five times for 1 s in the fixative solution, then three times for 1 s in both the solution I and in the solution II. Finally the glass slide was rinsed one time for 1 s in the solution III. Haemocytes were counted under the optical microscope (40× objective). The technique was repeated in an identical way in both experimental blocks. In Block 2, however, due to a problem with a one of the batches of the anticoagulant solution, the number of haemocytes in the young cohort could not be quantified.

Haemocytes were classified based on their morphological appearance into granulocytes, easily distinguishable by their ability to spread on glass surfaces, and oenocytoids, characterized by their spherical shape (see Additional file 2). According to Hillyer et al. [25] these are the only two types of circulating haemocytes capable of adhering to glass slides. A third population of haemocytes called prohaemocytes has also been identified in some studies [40]. Prohaemocytes are morphologically similar to oenocytoids, only smaller. In addition, their origin and functional role are still under discussion: they may be multipotent stem cells that give rise to the other haemocyte types [26] or may arise through the asymmetrical division of granulocytes [49]. As these prohaemocytes may constitute a very small fraction of the total number of circulating haemocytes [40] and since no clear size threshold existed to confidently distinguish between oenocytoids and prohaemocytes, no such distinction was made. It is thus worth bearing in mind that the population referred to as being oenocytoids may have included a small fraction of prohaemocytes.

### Statistical analyses

Analyses were carried out using the R statistical package (v. 3.1.1). The different statistical models built to analyse the data are described in Additional file 3. The analysis of response variables, which may depend on which bird the mosquitoes fed on, such oocyst prevalence and oocyst burden was carried out using mixed model procedures, fitting bird as a random factor in the models and treatment (young, old-unfed, old-blood fed) and blood meal size (haematin) as fixed factors. Oocyst prevalence (presence/absence of oocysts) was analysed using the *lmer* mixed model procedure and binomial errors, while oocyst burden (number of oocysts in individuals with more than one oocyst) was normalized using a logarithmic transformation and analysed using the *lme* procedure. All other variables were analysed using standard general linear models (glm) with an appropriate error distribution: wing size and haemocyte counts were analysed with a normal error distribution.

Maximal models, including all higher order interactions, were simplified by sequentially eliminating non-significant terms and interactions to establish a minimal model [50]. The significance of the explanatory variables was established using either a likelihood ratio test (which is approximately distributed as a Chi square distribution [51]) or an F test. The significant Chi square or F values given in the text are for the minimal model, whereas non-significant values correspond to those obtained before the deletion of the variable from the model. A posteriori contrasts were carried out by aggregating factor levels together and by testing the fit of the simplified model using a likelihood-ratio test [50].

### Ethical statement

Animal experiments were carried out in strict accordance with the National Charter on the Ethics of Animal Experimentation of the French Government, and all efforts were made to minimize suffering. Experiments were approved by the Ethical Committee for Animal Experimentation established by the authors’ institution (CNRS) under the auspices of the French Ministry of Education and Research (permit number CEEA- LR-1051).

## Results

### Haemocyte density

**Fig. 2:**
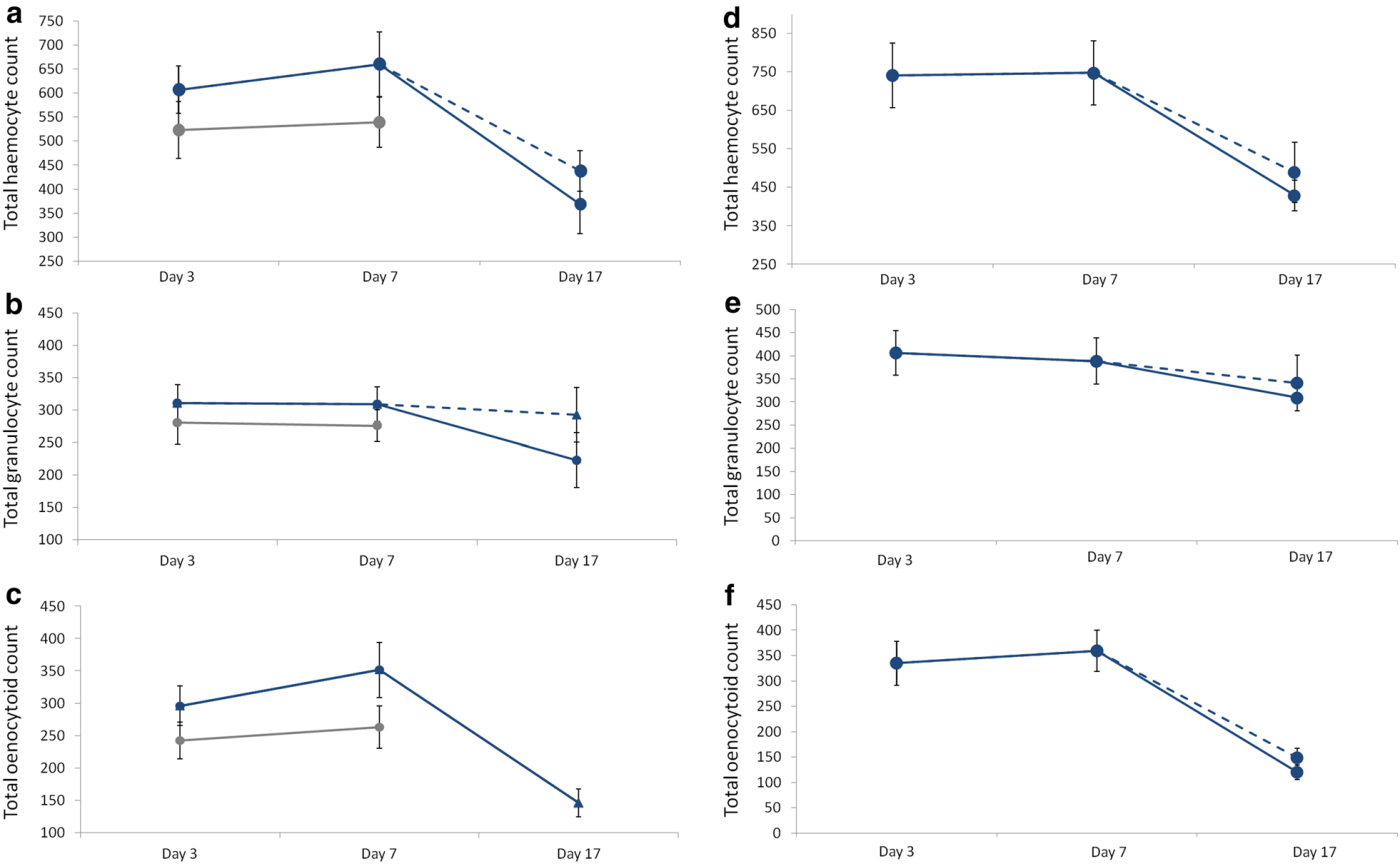
Haemocyte counts in the haemolymph of young (*grey lines*) and old (*blue lines*) mosquito cohorts across the three different time points since emergence in Block 1 (**a**, **b**, **c**) and Block 2 (**d**, **e**, **f**) of the experiment. Half of the mosquitoes in the old cohort were blood fed on Day 7 (see Fig. 1). Blood-fed females are represented by a dashed line, unfed females by a full line (when *dashed* and *full lines* overlap only the latter is shown). Total haemocytes (**a**, **d**) are the sum of the total number of granulocytes (**b**, **e**) and oenocytoids (**c**, **f**). Due to a problem with an anticoagulant solution, in Block 2 only the haemocytes of old-unfed and old-fed mosquitoes could be quantified

As there were no significant differences in size between the females in the different treatments (treatment effect, Block 1: model 5: F_1,73_ = 0.066, p = 0.417, Block 2: model 19: F_1,37_ = 0.089, p = 0.328), female size was not included in subsequent analyses. In Block 1, the old mosquito cohort was analysed to establish whether granulocytes, oenocytoids or the total haemocyte counts change with time. For this purpose, differences between old-unfed and old-blood fed females on Day 17 were first tested. As neither total haemocyte (model 6: F_1,19_ = 0.819 p = 0.377), granulocyte (model 9: F_1,19_ = 0.1582 p = 0.224) or oenocytoid numbers (model 12: F_1,19_ = 0.002 p = 0.999) differed between blood fed and unfed individuals (Fig. 2), the effect of time within the old mosquito cohort on these variables was carried out pooling these two data points together. This analysis showed that time has a significant effect on the total number of haemocytes (model 7: F_2,47_ = 8.007 p = 0.001, Fig. 2a) and oenocytoids (model 13: F_2,47_ = 20.820 p < 0.0001, Fig. 2c), but not on the number of granulocytes (model 10: F_2,47_ = 1.320 p = 0.277, Fig. 2b). While total haemocytes and oenocytoids remained stable at around 586 ± 25 and 325 ± 26 cells, respectively, between Days 3 and 7, their numbers decreased by ~40 % thereafter (contrast analyses Day 7 vs 17, total haemocytes: F_1,48_ = 13.793 p < 0.001, oenocytoids: F_1,48_ = 34.755 p < 0.0001). These analyses also revealed no significant effects of either treatment or time on the number of either total haemocytes, granulocytes or oenocytoids between the old and young females on Days 3 and 7, the time points shared by the two cohorts (interaction treatment × time effect, total haemocytes: model 8: F_2,50_ = 3.119, p = 0.083, granulocytes: model 11: F_2,50_ = 0.003, p = 0.956, oenocytoids: model 14: F_2,50_ = 0.034, p = 0.854, Fig. 2).

The results of Block 2 for the old-unfed and old-fed treatments (the haemocyte quantification for the young treatment could not be carried out due to a problem with one of the anticoagulant batches, see above) were highly consistent with those obtained in Block 1 (Fig. 2). As above, neither total haemocyte (model 20: F_1,16_ = 0.470, p = 0.503), granulocyte (model 22: F_1,16_ = 0.2284, p = 0.639) or oenocytoid numbers (model 24: F_1,16_ = 1.354, p = 0.262) differed between blood fed and unfed individuals so the data were pooled for subsequent analyses. Time has a significant effect on the total number of haemocytes (model 21: F_2,36_ = 6.891, p = 0.003, Fig. 2d) and oenocytoids (model 25: F_2,36_ = 17.716, p < 0.0001, Fig. 2f), but not on the number of granulocytes (model 23: F_2,36_ = 1.191, p = 0.315, Fig. 2e).

### *Plasmodium* prevalence and intensity

As there were no differences in size between the females in the three different treatments (treatment effect Block 1, model 1: *χ*_2_^2^ = 2.848, p = 0.609; Block 2, model 11: *χ*_2_^2^ = 2.881, p = 0.0747), female size was not included in subsequent analyses. The amount of blood ingested, however, varied significantly between the treatments (Block 1, model 2: *χ*_5_^2^ = 7.6836, p < 0.0001; Block 2, model 16: *χ*_5_^2^ = 41.851, p < 0.0001): young females took significantly larger blood meals than old-unfed (contrast analyses: Block 1: *χ*_4_^2^ = 14.988, p < 0.0001, Block 2: *χ*_4_^2^ = 69.009, p < 0.0001) and old-fed females (Block 1: *χ*_4_^2^ = 3.221, p = 0.073, Block 2: model 13: *χ*_4_^2^ = 32.622, p < 0.0001). Old-fed females also took larger blood meals than old-unfed ones (Block 1: *χ*_4_^2^ = 4.866, p = 0.027, Block 2: *χ*_4_^2^ = 13.106, p < 0.0001). As blood meal size can be a strong predictor of both the prevalence and the intensity of the infection, this explanatory variable was introduced into all subsequent analyses.

**Fig. 3:**
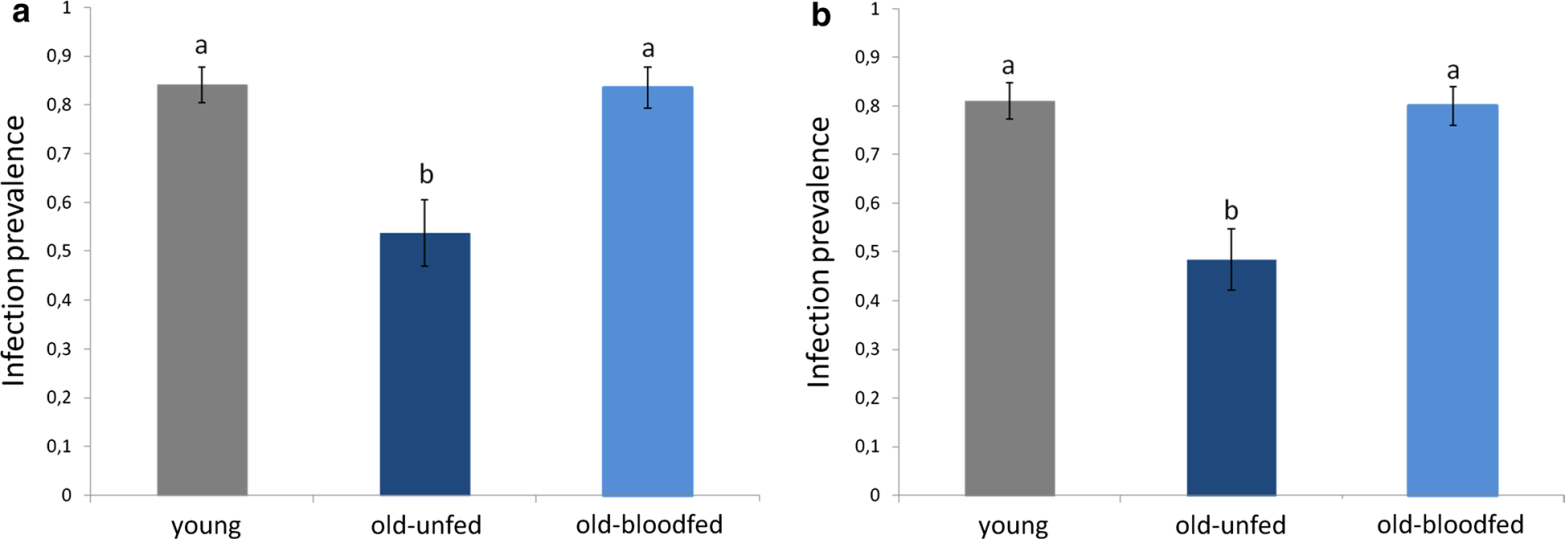
Infection prevalence (% mosquitoes containing ≥1 oocyst) in young (7-day old mosquitoes) and in old (17-day old) mosquitoes that were either allowed a previous uninfected blood meal (old-blood fed) or not. Figure represents results for Block 1 (**a**) and Block 2 (**b**)

The results of Blocks 1 and 2 are highly consistent in showing a strong effect of female age on the probability of becoming infected by *Plasmodium* (treatment effect Block 1, model 3: *χ*_5_^2^ = 13.08, *p* = 0.001; Block 2, model 17: *χ*_5_^2^ = 21.123, *p* < 0.001, Fig. 3a, b). In both blocks, the probability of infection of old females was a roughly a third lower than that of young females (contrast analyses young *vs* old-unfed Block 1: *χ*_4_^2^ = 9.433, *p* = 0.002; Block 2: *χ*_4_^2^ = 22.561, *p* < 0.001). However, taking an uninfected blood meal 10 days before the infected one reverses the protective effects of age so that the prevalence of infection in old-blood fed females was no different to that of young females (contrast analyses Block 1: *χ*_4_^2^ = 0.071, *p* = 0.790, Block 2 : *χ*_4_^2^ = 0.023, *p* = 0.880, Fig. 3a, b). Including haematin in the analyses did not alter the significance of these results.

**Fig. 4:**
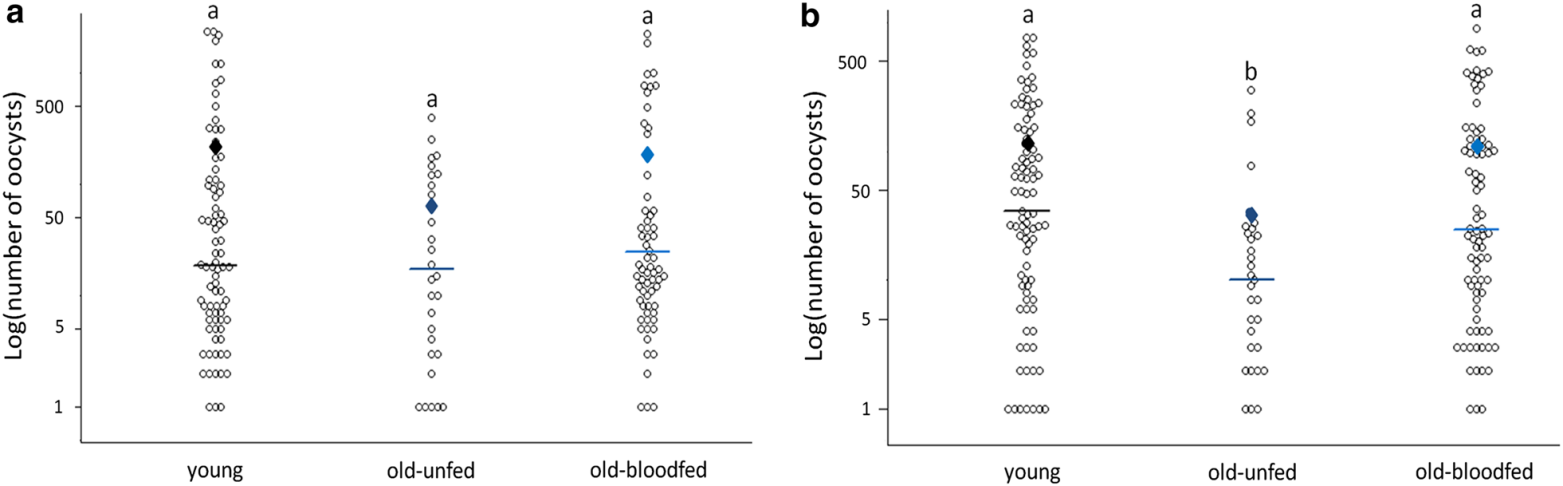
Mean oocyst burden (NB oocysts across mosquitoes containing ≥1 oocyst) in young (7-day old mosquitoes) and in old (17-day old) mosquitoes that were either allowed a previous uninfected blood meal (old-blood fed) or not. Figure represents results for Block 1 (**a**) and Block 2 (**b**)

A similar trend was observed in the intensity of the infection: old-unfed females have roughly three times fewer oocyst burdens than young and old-blood fed ones, although the results are only statistically significant in the second experimental block (treatment effect Block 2, model 18: *χ*_2_^2^ = 14.405, *p* < 0.001, Fig. 4b). Including haematin in the analyses did not alter the significance of these results. Block 1 followed a similar trend, with lower oocyst burdens in old-unfed females than in the other two treatments, but the results were not statistically significant (treatment effect Block 1, model 11: *χ*_2_^2^ = 3.607, *p* = 0.165, Fig. 4a). Here, however, a significant positive correlation between haematin and oocyst burden was found (haematin effect, model 11: *χ*_4_^2^ = 18.515, *p* < 0.001).

## Discussion

The results of these experiments show that: (1) haemocyte numbers decrease drastically as mosquitoes’ age; (2) this effect is not correlated with an increased susceptibility to *Plasmodium*, rather, older mosquitoes are significantly more resistant to malaria parasites than their younger counterparts; and, (3) the increased resistance with age is reversed when mosquitoes have previously taken an uninfected blood meal. Below each of these results separately are discussed separately.

### Immunity wanes with age

Immune defences tend to decline as individuals age. This decline may be the result of the generalized, and inevitable, process of physiological wear and tear, or an adaptive adjustment of immune function with age [12, 16]. Age-related declines in immune function have been documented in several insect species using a variety of immune indicators, such as phenoloxidase (PO) activity, encapsulation rate and haemocyte numbers [25, 52]. The results of the current experiments show that 17-day old *Cx pipiens* mosquitoes have 40 % fewer haemocytes than their younger (7-day old) counterparts. Interestingly, this decline in haemocytes with time is entirely due to a drastic decrease in the number of oenocytoids, while granulocytes remain largely unchanged. Granulocytes and oenocytoids play different roles in the immune system. Granulocytes primarily kill pathogens through phagocytosis or lysis, while oenocytoids are the major producers of the enzymes required for melanization, including PO [40]. The drastic decrease in oenocytoids observed agrees with previous results showing that PO activity declines drastically with age so that, by Day 14, *Cx pipiens* females have lost around two-thirds of their PO at emergence [27]. Immune decay with age has also been observed in the two other major mosquito vector species, *Ae. aegypti* and *Anopheles gambiae*, where decreases in haemocyte numbers [25, 26] and melanization potential [23, 24] have been documented. Taking a previous blood meal did not alter the course of immune senescence in mosquitoes. Previous work has shown that blood feeding activates the mosquito innate immune system [26, 53, 54]. The effect is however transient, which probably explains why no differences in mosquitoes were found 10 days after the uninfected blood meal.

### Susceptibility to *Plasmodium* decreases with age

Immune decay has led to predictions that as insects age their ability to clear infections should also decrease [55, 56]. The results of the current experiments contradict this prediction. Despite a drastic decrease in the immune system with age, old *Cx pipiens* females are significantly more resistant to a *P. relictum* infection than younger ones. The probability of becoming infected by *P. relictum* is 30 % lower in 17-day old than in seven-day old mosquitoes. These results are consistent across two different experimental blocks carried out several weeks apart under identical experimental conditions. Two other studies to date have investigated the role of mosquito age on *Plasmodium* infection. Terzian et al. [37], who carried out what is probably the first investigation into the effect of insect age on susceptibility to infection in any invertebrate, also reported that as *Ae. aegypti* age they become more resistant to a *P. gallinaceum* infection. More recently, however, Okech et al. [57] found no effect of age on the prevalence of *Plasmodium falciparum* infections in *An. gambiae*, although the low infection rates in these experiments (<4 % of the membrane-fed mosquitoes became infected) may have significantly reduced the statistical power of their experiments.

Interestingly, a decrease in the probability of infection with age seems to be a common feature in insects (see Additional file 1). It has been reported in bacteria-challenged drosophila flies [58], *Trypanosoma*-challenged tsetse flies [59], *Microsporidium*-challenged bees [60, 61] and virus and *Filaria*-challenged mosquitoes [43, 62, 63]. The mechanisms underlying these increased susceptibilities to infection later in life are not known, but none of these studies measured immunity, so a re-allocation of resources towards a particular arm of the immune system cannot be entirely excluded [16]. Unfortunately, only a handful of studies have concomitantly measured immunity and parasite infection rates in aging insects. Hillyer et al. [25] found that a decrease in haemocyte counts with age increases the susceptibility of *Ae. aegypti* mosquitoes to injected *Escherischia coli* bacteria. Similarly, Roberts and Hughes [28] found that a decrease in PO level in older bumblebees was associated with increased microsporidian infection rates. Clearly, more studies are needed that investigate the effect of age not only on immunity but also on parasite infection rates since, as the results presented here show, both are not necessarily correlated.

The fact that a decrease in PO [27] and PO-producing oenocytoids (Fig. 2) does not result in an increase in *Plasmodium* infection rates could be partly explained by PO playing no role in *Plasmodium* protection in this system. Current knowledge of the role of PO in *Plasmodium* protection is largely based on studies using genetically selected resistant and susceptible mosquitoes and gene-silencing procedures [3] but there is hardly any evidence that melanization plays a role in defence against *Plasmodium* in the field (e.g., [24]), and *P. relictum* oocysts in *Cx pipiens* mosquitoes are rare. Melanization, however, is the most apparent, but not the only, outcome of the PO cascade. The PO enzymatic cascade also produces many cytotoxic intermediates that generate reactive oxygen and nitrogen intermediates [64] which may kill the parasite without encapsulating it [65]. Alternatively, the possibility that the decrease in haemocyte counts and PO activity with age in *Cx pipiens* mosquitoes is correlated with the upregulation of another unmeasured, immune effector, cannot be entirely excluded. Potential candidates are antimicrobial peptides, which are produced by the mosquito fat body [25] so their titers may be uncorrelated with haemocyte densities [16]. Indeed, Moret and Schmid-Hempel [16] found that, as bumblebees age, a decrease in haemocyte numbers and PO activity was associated with an increase in antimicrobial activity. However, Lambrechts et al. [66] however, found a positive correlation between PO activity and antibacterial defence. In addition, previous results showing that insecticide-resistant *Cx pipiens* mosquitoes show no differences in *P. relictum* prevalence or intensity, despite having significantly higher levels of antimicrobial peptide expression [46, 67], suggest that these peptides may not play a key role in *Plasmodium* defence in this system. It is thus highly likely that immunity is not directly responsible for the lower infection rates observed in old mosquitoes and that an explanation must therefore be found elsewhere.

The journey of *Plasmodium* through the mosquito requires the complex interplay of a suite of finely tuned physiological, immunological and molecular events, many of which take place in the mosquito midgut [68, 69]. In recent years, the role played by the different arms of the mosquito immune system in limiting *Plasmodium* development has received a great deal of attention (recently reviewed by Clayton et al. [3]). Fewer efforts have been directed at investigating how other physiological events, which are essential for the successful transformation and passage of the different *Plasmodium* stages through the mosquito, vary across individuals and experimental conditions. Most of these events take place in the mosquito midgut and include: the production of large amounts of xanthurenic acid, a by-product of the synthesis of the eye pigment, which is essential for the exflagellation of gametocytes in the midgut [70], the secretion of digestive trypsin-like enzymes which aid ookinetes to break down and traverse the chitinous peritrophic membrane [71], the binding of ookinetes to specific midgut epithelial ligands [69] and to structural proteins in the basal lamina [72], and the secretion of digestive carboxypeptidase enzymes which may provide *Plasmodium* with essential amino acids for oocyst development [68]. It is likely that some of these factors are disturbed as mosquitoes age. Recent work has indeed shown that in *Drosophila,* ageing is accompanied by both a structural and a functional degeneration of the fly’s midgut [73]. In particular, a decrease in trypsin production with age has been observed not only in *Drosophila* [73], but also in *Anopheles* [74]. Equivalent changes in the *Culex* midgut could explain the decrease in *Plasmodium* infection with age observed in this system. In addition, recent work has demonstrated the crucial role played by the midgut microbial flora in the success of *Plasmodium* infections in mosquitoes [75–77]. In flies, the number of micro-organisms found in the lumen of the gut increases significantly with age [78]. Further work is needed on how the gut microbiota changes as mosquito ages and on the consequences this may have for *Plasmodium* transmission.

### A prior blood meal reverses the effect of age on *Plasmodium* susceptibility

The fact that the age effect is reversed when old mosquitoes have taken one previous non-infected blood meal is significant and may provide further clues about the mechanisms underlying the effect of mosquito age on infection. Interestingly, Terzian [37] and Ariani et al. [43] also found that a previous blood meal reverses the negative effect of age on the development of two very different parasites: *Plasmodium* and the filarial nematode *Brugia malayi*, respectively. Both speculated that a long stretch without a blood meal may have depleted the older females of key nutrients required for parasite development, and that the additional blood meal may have provided the extra resources. Terzian et al. [37] explored the possibility that nitrogen or protein depletion were responsible for their results by substituting the first blood meal with a diet of chicken plasma and haemoglobin. Puzzlingly, this diet resulted in an even larger decrease in *Plasmodium* oocyst numbers than in sugar-fed mosquitoes. Complementing the diet with a raisin infusion did, however, restore oocyst numbers to those obtained in younger mosquitoes [37]. Clearly, more work is needed on the nutritional needs of *Plasmodium* inside the mosquito and on whether key limiting nutrients for *Plasmodium* development are acquired by the mosquito during their adult or larval lives [79].

Aside from their nutritional contents, blood meals also trigger a whole set of physiological events in the mosquito, which could impact the infection success of a subsequent infected blood meal. Taking a warm blood meal induces a temperature stress that results in the synthesis of *hsp70* [80], a heat shock protein that has been shown to lead to drastic increases in oocyst burdens in *Plasmodium*-infected *Anopheles* [81]. In addition, ingestion of a first blood meal may alter the kinetics of digestion of subsequent blood meals [82]. In *An. gambiae,* ingestion of a prior blood meal accelerates the digestive process of subsequent infected blood meals, thereby reducing the probability of a *Plasmodium* infection [82], while in *Ae. aegypti*, subsequent digestions are slowed down [83]. Because parasite fertilization, transformation and penetration through the midgut require time, a slower blood meal digestion in previously blood-fed *Cx pipiens* females would have afforded the parasite more time to establish a successful infection in the mosquito [82].

## Conclusion

Mosquito populations are structured both by age and by the time since they took their infectious blood meal [35]. The results of these experiments show that age significantly impacts the susceptibility of mosquitoes to a *Plasmodium* infection: older females have a lower probability of becoming infected with the parasite. However, the dramatic effects of age are reversed in previously blood-fed females. This point is crucial because most old mosquitoes in the field are likely to have taken at least one previous blood meal. Indeed, up to 80 % of malaria vectors collected in the field are parous: i.e., have taken a blood meal and laid a batch of eggs (e.g., [84, 85]). As a consequence, in the field, age is unlikely to have a significant impact on the susceptibility of mosquitoes to a malaria infection. However, investigating the mechanisms underlying both the increased resistance of old unfed mosquitoes, and its reversal when mosquitoes are previously given blood meal, may unravel key insights about the biology and development of *Plasmodium* parasites in the mosquito.

## Additional files

10.1186/s12936-015-0912-z Impact of age on parasite prevalence and parasite intensity in experimentally infected insects, and correlated measurements of immune senescence, when available.
10.1186/s12936-015-0912-z Optical microscope (x400) image of *Cx pipiens* haemocytes showing the two different morphotypes described in this study: granulocytes and oenocytoids (see main text for details).
10.1186/s12936-015-0912-z Description of statistical models used to analyse the data.
