# Supplementary material for: Mosquito age and avian malaria infection
Source: Malar J. 2015 Sep 30;14:383. doi: 10.1186/s12936-015-0912-z (PMC4589955; doi:10.1186/s12936-015-0912-z)
Supplement: Supplementary file 1 — 10.1186/s12936-015-0912-z Impact of age on parasite prevalence and parasite intensity in experimentally infected insects, and correlated measurements of immune senescence, when available. [file 12936_2015_912_MOESM1_ESM.docx]

**Additional file 1: Impact of age on parasite prevalence and parasite intensity in experimentally infected insects, and correlated measurements of immune senescence, when available**. WNV: West Nile Virus*,* SLE: Saint Louis Encephalitis Virus, JEV: Japanese Encephalitis Virus, ×: not reported. Superscripts: ^1^immune senescence is quantified in uninfected individuals, ^2^the age effect differs between genetic lines, ^3^the age effect depends on temperature and virus dose, ^4^the age effect depends on the immune parameter measured

|  | **Age range** | **Parasite** | **Parasite**  **prevalence** | **Parasite**  **intensity** | **Immune senescence^1^** | **REF** |
| --- | --- | --- | --- | --- | --- | --- |
| **Flies** |  |  |  |  |  |  |
| *D. melanogaster* | 3-13 days | bacteria (*S. marescens*) | × | **decreases** | × | [1] |
| *D. melanogaster* | 8-75 days | bacteria (several species) | × | no effect | × | [2] |
| *D. melanogaster* | 3-40 days | bacteria (*E. coli*) | × | no effect | × | [3] |
| *D. melanogaster* | 1-4 weeks | bacteria (*E. coli*) | × | **decreases** / increases^2^ | × | [4] |
| *M. domestica* | 1-14 days | fungus (*B. basana*) | no effect | × | × | [5] |
| *G. morsitans* | 24-48 hours | trypanosome (*T. brucei*) | **decreases** | × | × | [6] |
| *G. morsitans* | 24-48 hours | trypanosome (*T. congolense*) | **decreases** | × | × | [6] |
| *G. palpalis* | 24-48 hours | trypanosome (*T. brucei*) | **decreases** | × | × | [6] |
| **Mosquitoes** |  |  |  |  |  |  |
| *C. pipiens* | 4-10 days | virus (WNV) | **decreases** / increases^3^ |  | × | [7] |
| *C. pipiens* | 3-12 days | virus (SLEV) | **decreases** | × | × | [8] |
| *C. pipiens* | 2-22 days | filaria (*Brugia sp.*) | increases | × | × | [9] |
| *C. tritaeniorhynchus* | 10-24 days | virus (JEV) | no effect | × | × | [10] |
| *C. tritaeniorhynchus* | 4-12 days | virus (WNV) | **decreases** | × | × | [11] |
| *A. aegypti* | 1-5 days | bacteria (*E. coli*) | × | increases | **yes** | [12] |
| *A. aegypti* | 1-6 weeks | filaria (*B. malayi*) | **decreases** | **decreases** | × | [13] |
| *A. aegypti* | 1-4 weeks | apicomplexa (*P. gallinaceum*) | × | **decreases** | × | [14] |
| *A. gambiae* | 2-12 days | fungus (*M. anisopliae*) | × | no effect | × | [15] |
| *A. gambiae* | 1-11 days | apicomplexa (*P. falciparum*) | no effect | no effect | × | [16] |
| **Bees** |  |  |  |  |  |  |
| *A. mellifera* | 1-14 days | microsporidia (*N. ceranae*) | × | increases | **yes** / no^4^ | [17] |
| *A. mellifera* | 1-12 days | microsporidia (*N. ceranae*) | **decreases** | **decreases** | **yes** / no^4^ | [18] |
| *B. terrestris* | 2-10 | microsporidia (*N. bombi*) | **decreases** | × | × | [19] |
|  |  |  |  |  |  |  |

**References:**

1. Khan I, Prasad NG: The aging of the immune response in *Drosophila melanogaster*. J Gerontol A Biol Sci Med Sci 2013, 68:129–135.

2. Ren C, Webster P, Finkel SE, Tower J: Increased internal and external bacterial load during *Drosophila* aging without life-span trade-off. Cell Metab 2007, 6:144–152.

3. Ramsden S, Cheung YY, Seroude L: Functional analysis of the *Drosophila* immune response during aging. Aging Cell 2008, 7:225–236.

4. Lesser KJ, Paiusi IC, Leips J: Naturally occurring genetic variation in the age-specific immune response of *Drosophila melanogaster*. Aging Cell 2006, 5:293–295.

5. Kaufman PE, Wood LA, Goldberg JI, Long SJ, Rutz DA: Host age and pathogen exposure level as factors in the susceptibility of the house fly, *Musca domestica* (*Diptera: Muscidae*) to *Beauveria bassiana*. Biocontrol Sci Technol 2008, 18:841–847.

6. Walshe DP, Lehane MJ, Haines LR: Post eclosion age predicts the prevalence of midgut trypanosome infections in *Glossina*. PLoS ONE 2011, 6:e26984.

7. Richards SL, Lord CC, Pesko K, Tabachnick WJ: Environmental and biological factors influencing *Culex pipiens quinquefasciatus* say (*Diptera: Culicidae*) vector competence for Saint Louis Encephalitis Virus. Am J Trop Med Hyg 2009, 81:264–272.

8. Richards SL, Lord CC, Pesko KN, Tabachnick WJ: Environmental and biological factors influencing *Culex pipiens quinquefasciatus* (*Diptera: Culicidae*) vector competence for West Nile Virus. Am J Trop Med Hyg 2010, 83:126–134.

9. Desowitz RS, Chellappah WT: The transmission of *Brugia* *sp*. through *Culex pipiens fatigans*: The effect of age and prior non-infective blood meals on the infection rate. Trans R Soc Trop Med Hyg 1962, 56:121–125.

10. Takahashi M: The effects of environmental and physiological conditions of *Culex tritaeniorhynchus* on the pattern of transmission of Japanese encephalitis virus. J Med Entomol 1976, 13:275–284.

11. Baqar S, Hayes CG, Ahmed T: The effect of larval rearing conditions and adult age on the susceptibility of *Culex tritaenio-rhynchus* to infection with West Nile virus. Mosq News 1980, 40:165–171.

12. Hillyer JF, Schmidt SL, Fuchs JF, Boyle JP, Christensen BM: Age-associated mortality in immune challenged mosquitoes (*Aedes aegypti)* correlates with a decrease in haemocyte numbers. Cell Microbiol 2005, 7:39–51.

13. Ariani CV, Juneja P, Smith S, Tinsley MC, Jiggins FM: Vector competence of *Aedes aegypti* mosquitoes for filarial nematodes is affected by age and nutrient limitation. Exp Gerontol 2015, 61:47–53.

14. Terzian LA, Stahler N, Irreverre F: The effects of aging, and the modifications of these effects, on the immunity of mosquitoes to malarial infection. J Immunol 1956, 76:308–313.

15. Mnyone LL, Kirby MJ, Mpingwa MW, Lwetoijera DW, Knols BGJ, Takken W, Koenraadt CJM, Russell TL: Infection of *Anopheles gambia*e mosquitoes with entomopathogenic fungi: effect of host age and blood-feeding status. Parasitol Res 2011, 108:317–322.

16. Okech BA, Gouagna LC, Kabiru EW, Beier JC, Yan G, Githure JI: Influence of age and previous diet of *Anopheles gambiae* on the infectivity of natural *Plasmodium falciparum* gametocytes from human volunteers. J Insect Sci 2004, 4.

17. Roberts KE, Hughes WOH: Immunosenescence and resistance to parasite infection in the honey bee, *Apis mellifera*. J Invertebr Pathol 2014, 121:1–6.

18. Chaimanee V, Chantawannakul P, Chen Y, Evans JD, Pettis JS: Effects of host age on susceptibility to infection and immune gene expression in honey bee queens (*Apis mellifera*) inoculated with *Nosema ceranae*. Apidologie 2013, 45:451–463.

19. Rutrecht ST, Klee J, Brown MJF: Horizontal transmission success of *Nosema bombi* to its adult bumble bee hosts: effects of dosage, spore source and host age. Parasitology 2007, 134(Pt 12):1719–1726.
