# Supplementary material for: Mosquito age and avian malaria infection
Source: Malar J. 2015 Sep 30;14:383. doi: 10.1186/s12936-015-0912-z (PMC4589955; doi:10.1186/s12936-015-0912-z)
Supplement: Supplementary file 2 — 10.1186/s12936-015-0912-z Optical microscope (x400) image of Cx pipiens haemocytes showing the two different morphotypes described in this study: granulocytes and oenocytoids (see main text for details). [file 12936_2015_912_MOESM2_ESM.docx]

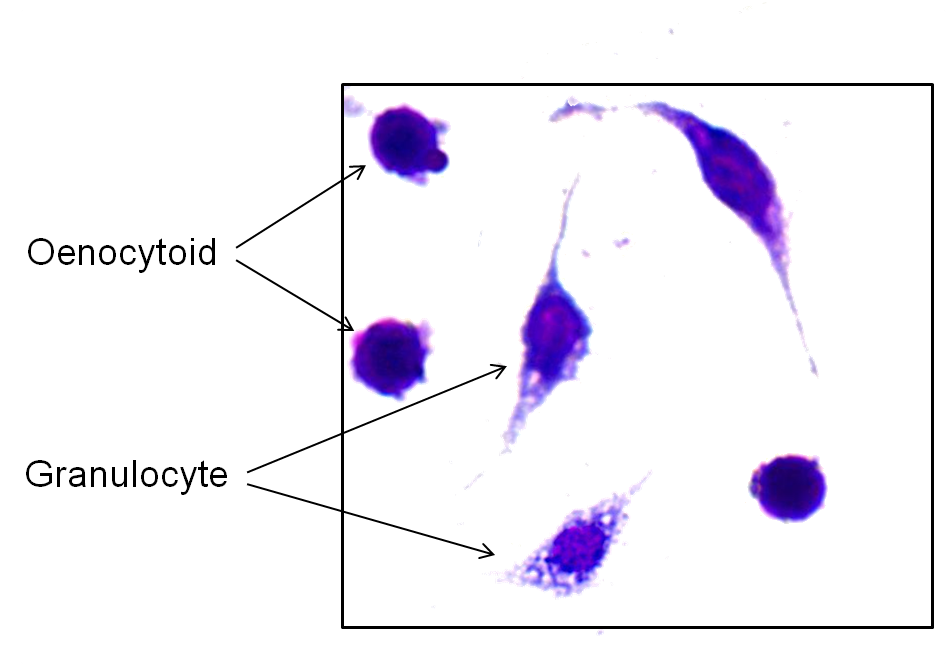


**Additional file 2**: Optical microscope (x400) image of *Cx pipiens* haemocytes showing the two different morphotypes described in this study: granulocytes and oenocytoids (see main text for details).
