# Supplementary material for: Mosquito age and avian malaria infection
Source: Malar J. 2015 Sep 30;14:383. doi: 10.1186/s12936-015-0912-z (PMC4589955; doi:10.1186/s12936-015-0912-z)
Supplement: Supplementary file 3 — 10.1186/s12936-015-0912-z Description of statistical models used to analyse the data. [file 12936_2015_912_MOESM3_ESM.docx]

**Additional file 3:** **Description of statistical models used to analyze the data.** The response variable was not transformed unless otherwise stated. N gives the number of mosquitoes included in each analysis. "Maximal model" gives the complete set of explanatory variables (and their interactions) included in the model. "Minimal model" gives the model containing only the significant variables and their interactions. Round brackets indicate variables fitted as random factors (1/bird denotes that the effect of bird is fitted as a random variable). Square brackets indicate the error structure used (n: normal, b: binomial). treat: treatment ("young", "old-unfed", "old-bloodfed"), hm: haematin excreted (blood meal size), time: (time since adult emergence: D3, D7 or D17) .

| **Variable of interest** | **Response**  **variable** | **Model**  **nb** | **N** | **Maximal model** | **Minimal model** | **R subrout**  **[error]** |
| --- | --- | --- | --- | --- | --- | --- |
| **Block 1** |  |  |  |  |  |  |
| *Plasmodium prevalence and burden* |  |  |  |  |  |  |
| Wing size | size | 1 | 229 | treat | 1 | glm [n] |
| Blood meal size | hm | 2 | 229 | treat + (1/bird) | treat + (1/bird) | lme [n] |
| Oocyst prevalence | prev | 3 | 229 | treat*hm + (1/bird) | treat + hm + (1/bird) | lmer [b] |
| Oocyst burden | log (burd) | 4 | 229 | treat*hm + (1/bird) | hm + (1/bird) | lme [n] |
| *Haemocyte density* |  |  |  |  |  |  |
| Wing size | size | 5 | 75 | treat | 1 | glm [n] |
| Nb total haemocytes, day 17 | hct | 6 | 21 | treat | 1 | glm [n] |
| Nb total haemocytes through time ("old" cohort) | hct | 7 | 50 | time | time | glm [n] |
| Nb total haemocytes, days 3 and 7 | hct | 8 | 54 | treat*time | 1 | glm [n] |
| Nb granulocytes, day 17 | grn | 9 | 21 | treat | 1 | glm [n] |
| Nb granulocytes through time ("old" cohort) | grn | 10 | 50 | time | 1 | glm [n] |
| Nb granulocytes, days 3 and 7 | grn | 11 | 54 | treat*time | 1 | glm [n] |
| Nb oenocytoids, day 17 | log (oen) | 12 | 21 | treat | 1 | glm [n] |
| Nb oenocytoids through time ("old" cohort) | log (oen) | 13 | 50 | time | time | glm [n] |
| Nb oenocytoids, days 3 and 7 | log (oen) | 14 | 54 | treat*time | 1 | glm [n] |
| **Block 2** |  |  |  |  |  |  |
| *Plasmodium prevalence and burden* |  |  |  |  |  |  |
| Wing size | size | 15 | 271 | treat | 1 | glm [n] |
| Blood meal size | hm | 16 | 271 | treat +(1/bird) | treat +(1/bird) | lme [n] |
| Oocyst prevalence | prev | 17 | 271 | treat*hm + (1/bird) | treat*hm + (1/bird) | lmer[b] |
| Oocyst burden | log (burd) | 18 | 271 | treat*hm + (1/bird) | treat*hm + (1/bird) | lme [n] |
| *Haemocyte density* |  |  |  |  |  |  |
| Wing size | size | 19 | 39 | treat | 1 | glm [n] |
| Nb total haemocytes, day 17 | hct | 20 | 18 | treat | 1 | glm [n] |
| Nb total haemocytes through time ("old" cohort) | hct | 21 | 39 | time | time | glm [n] |
| Nb granulocytes, day 17 | grn | 22 | 18 | treat | 1 | glm [n] |
| Nb granulocytes through time ("old" cohort) | grn | 23 | 39 | time | 1 | glm [n] |
| Nb oenocytoids, day 17 | log (oen) | 24 | 18 | treat | 1 | glm [n] |
| Nb oenocytoids through time ("old" cohort) | log (oen) | 25 | 39 | time | time | glm [n] |
